# Autophagy, EVs, and Infections: A Perfect Question for a Perfect Time

**DOI:** 10.3389/fcimb.2018.00362

**Published:** 2018-10-18

**Authors:** Michelle L. Pleet, Heather Branscome, Catherine DeMarino, Daniel O. Pinto, Mohammad Asad Zadeh, Myosotys Rodriguez, Ilker Kudret Sariyer, Nazira El-Hage, Fatah Kashanchi

**Affiliations:** ^1^Laboratory of Molecular Virology, School of Systems Biology, George Mason University, Manassas, VA, United States; ^2^Department of Immunology, Herbert Wertheim College of Medicine, Florida International University, Miami, FL, United States; ^3^Department of Neuroscience, Center for Neurovirology, Lewis Katz School of Medicine, Temple University, Philadelphia, PA, United States

**Keywords:** autophagy, exosome, extracellular vesicle, virus, infectious disease, secretory autophagy

## Abstract

Autophagy, a highly conserved process, serves to maintain cellular homeostasis in response to an extensive variety of internal and external stimuli. The classic, or canonical, pathway of autophagy involves the coordinated degradation and recycling of intracellular components and pathogenic material. Proper regulation of autophagy is critical to maintain cellular health, as alterations in the autophagy pathway have been linked to the progression of a variety of physiological and pathological conditions in humans, namely in aging and in viral infection. In addition to its canonical role as a degradative pathway, a more unconventional and non-degradative role for autophagy has emerged as an area of increasing interest. This process, known as secretory autophagy, is gaining widespread attention as many viruses are believed to use this pathway as a means to release and spread viral particles. Moreover, secretory autophagy has been found to intersect with other intracellular pathways, such as the biogenesis and secretion of extracellular vesicles (EVs). Here, we provide a review of the current landscape surrounding both degradative autophagy and secretory autophagy in relation to both aging and viral infection. We discuss their key features, while describing their interplay with numerous different viruses (i.e. hepatitis B and C viruses, Epstein-Barr virus, SV40, herpesviruses, HIV, chikungunya virus, dengue virus, Zika virus, Ebola virus, HTLV, Rift Valley fever virus, poliovirus, and influenza A virus), and compare secretory autophagy to other pathways of extracellular vesicle release. Lastly, we highlight the need for, and emphasize the importance of, more thorough methods to study the underlying mechanisms of these pathways to better advance our understanding of disease progression.

## Introduction to autophagy

The discovery of the lysosome in 1955 by Christian de Duve was a landmark in the study of intracellular protein degradation (Ohsumi, 2014). Consequently, it was also de Duve who first used the term “autophagy,” or “self-eating” to define the phenomenon by which cytoplasmic components were digested by “autolytic vacuoles or cytolysomes,” which he reasoned were lysosomes due to their lytic activity (de Duve et al., 1955; de Duve, 1964). While it has been over fifty years since autophagy was first described, recent decades have experienced a significant increase in autophagy-related research. This interest was undoubtedly spurred in the early 1990's by Tsukada and Ohsumi's identification of the autophagy-related genes (ATGs) in yeast; an achievement for which Yoshinori Ohsumi was awarded the Nobel Prize in 2016 for Physiology and Medicine (Tsukada and Ohsumi, 1993; Münz, 2017).

Autophagy is a highly conserved pathway among eukaryotes that involves the recognition, capture, and trafficking of various intracellular components to the lysosome for degradation (He and Klionsky, 2009; Bento et al., 2016). In the most primitive sense, autophagy is responsible for maintaining cellular homeostasis. This is especially critical during periods of stress and starvation; under these conditions the coordinated breakdown of macromolecules via autophagy machinery provides key nutrients and energy to the cell, which are required to maintain viability (White et al., 2015; Bento et al., 2016). In the context of nutrient recycling, autophagy is largely considered to be non-selective, meaning that cytoplasmic components are randomly engulfed and processed for degradation. However, advanced studies have also demonstrated that autophagy can mediate the removal of specific intracellular substrates, such as misfolded proteins and damaged organelles (Gatica et al., 2018). This process has been termed selective autophagy and many different forms have been classified based on their cytosolic target. Examples of selective autophagy targets include mitochondria (mitophagy), the nucleus (nucleophagy), the endoplasmic reticulum (reticulophagy), lysosomes (lysophagy), and intracellular pathogens (xenophagy) (Ashrafi and Schwarz, 2013; Hung et al., 2013; Nakatogawa and Mochida, 2015; Anding and Baehrecke, 2017; Gatica et al., 2018). Due to the extremely diverse and specialized roles of organelles, it is imperative for the cell to monitor and regulate their number and health. The selective removal of defective or excessive organelles protects the cell from the buildup of toxic byproducts and, furthermore, is crucial for the regulation of homeostasis. While the underlying mechanisms are not yet fully understood, organelle clearance is believed to involve a cellular tag (i.e., ubiquitination) that marks the organelle for subsequent recognition and destruction (Anding and Baehrecke, 2017). Thus, autophagy acts both non-selectively and selectively to promote cell survival through nutrient recycling and to perform quality control activities in the cytoplasm. Additionally, autophagy has been noted as an important pathway for the processing and presentation of various molecules through major histocompatibility complex (MHC) class proteins, especially in antigen presenting cells and in the context of xenophagy (Crotzer and Blum, 2009).

Three major autophagic pathways have been described in mammals: microautophagy, chaperone-mediated autophagy (CMA), and macroautophagy (Parzych and Klionsky, 2014). Although there are features that are unique to each pathway, such as the type of cargo and the mode of cargo delivery, each concludes in the lysosome with the breakdown and reprocessing of the delivered material. In microautophagy intracellular substrates are engulfed from the cytoplasm via lysosomal membrane invaginations (Sahu et al., 2011). Alternatively, CMA involves chaperone proteins that selectively recognize target substrates and shuttle them to the lysosomal membrane for uptake and degradation. Heat shock cognate protein 70 (HSC70) is a major cytosolic chaperone that identifies targets that contain a unique consensus motif, KFREQ, and traffics them to a specific lysosomal membrane receptor (LAMP2A) (Dice, 1990; Bhattacharya and Eissa, 2015). In macroautophagy, henceforth referred to as autophagy, degradation of substrates results from a series of sequential steps that are carefully regulated. Initiation of macroautophagy occurs with the formation of double-membraned structures called an autophagasomes, which engulfs various cytoplasmic substrates and subsequently fuses with the lysosome to release its contents (Bento et al., 2016).

The process of autophagy is tightly controlled by a set of ATG proteins. These proteins are further regulated by several mechanisms that sense energy, stress, and nutrient levels within the cell (White et al., 2015). Although ATGs were originally discovered in yeast, many of their mammalian orthologs have since been identified (Bento et al., 2016). The canonical autophagy pathway consists of a series of sequential steps which include initiation, nucleation, elongation, and fusion (Bhattacharya and Eissa, 2015). Each step of this pathway is mediated by specific multi-protein complexes. The UNC-51-like-kinase I (ULK) complex (composed of ULK1, FIP200, ATG13, and ATG101) is responsible for the *de novo* formation of the cup-shaped, double-membrane autophagasome during the initiation stage (Jung et al., 2009; Ktistakis and Tooze, 2016). The autophagasome forms at a site called the phagophore, which, interestingly, is a topic of active debate amongst researchers due to the uncertainty of the intracellular origin of this structure (Russell et al., 2014). In fact, the autophagosomal membranes are thought to arise from a wide assortment of recycled cellular membranes from the ER, mitochondria, plasma membrane, and endosomes (Juhasz and Neufeld, 2006; Puri et al., 2013; Bento et al., 2016; Ktistakis and Tooze, 2016). The class III phosphatidylinositol-3-OH-kinase (PI(3)K) complex (composed of Beclin-1, VPS15, VPS34, and ATG14) is primarily associated with nucleation and is recruited to the growing autophagasome by the activation of the ULK complex. PI(3)K produces phosphatidylinositol-3-phosphate, which subsequently recruits another set of effector proteins to further drive membrane development. Two ubiquitin-like (UBL) systems, ATG12-ATG5 and ATG8-LC3, are responsible for the elongation phase, during which cytoplasmic components are engulfed by the expanding autophagasome (Mizushima et al., 1998; Sakoh-Nakatogawa et al., 2013; Bhattacharya and Eissa, 2015; Bento et al., 2016). The maturation and closure of the autophagosome is followed by its trafficking, via the microtubule network, to the lysosome. Fusion with the lysosome, a mechanism which is thought to be regulated via Rab7 and various SNARE proteins, results in the formation of the autolysosome (Jordens et al., 2001; Fader et al., 2009; Huotari and Helenius, 2011; Itakura et al., 2012; Hyttinen et al., 2013). Beclin-1 is also involved in this fusion stage. The last step, in which the contents of the autolysosome are digested, is performed by a diverse set of hydrolytic enzymes within the lysosome. In contrast to the pathway outlined above, recent studies have described noncanonical pathways in which ATG proteins can participate in pathways other than autophagy or autophagy proceeds without utilizing the core ATG proteins (Sanjuan et al., 2007; Zhu et al., 2007). For example, autophagy is now believed to play a role in the secretion of certain cytosolic materials. This non-degradative pathway is referred to as secretory autophagy and is currently an active area of research (Ponpuak et al., 2015; Ktistakis and Tooze, 2016), which will be discussed later.

As mentioned above, autophagy is upregulated in response to nutrient deprivation and other cellular stressors such as damaged organelles, oxidative stress, and infection (Bhattacharya and Eissa, 2015). Accordingly, many molecules and signaling cascades are involved in this response. In particular, mammalian target of rapamycin complex 1 (mTORC1) and AMP-activated protein kinase (AMPK) are key upstream regulators of the autophagy pathway due to their involvement in metabolite and energy sensing, respectively (Russell et al., 2014). The mTORC1 is a highly potent and strictly conserved inhibitor of autophagy. In mammals, mTORC1 has been shown to interact with the subunits of the ULK complex. When nutrients are abundant, mTORC1 phosphorylates ULK1 and ATG13, rendering the ULK complex unable to interact with other regulators, and thereby inhibiting the initiation of autophagy (Kim et al., 2011; Bento et al., 2016). Intracellular levels of energy must also be closely monitored to maintain homeostasis. Imbalances between energy generation and expenditure are sensed by AMPK, which can be activated in response to these imbalances. AMPK negatively regulates mTORC1 to suspend its repression of autophagy. This can occur either directly through the phosphorylation of the mTORC1 raptor subunit or indirectly through the phosphorylation of TSC, a complex which, when activated, represses mTORC1 (Inoki et al., 2003; Shaw et al., 2004; Russell et al., 2014). AMPK can also phosphorylate and activate the ULK1 complex directly (Bach et al., 2011; Kim et al., 2011). The net outcome of these actions, which result from the sensing of diminished energy levels within the cell, is the induction of autophagy. In addition to mTORC1 and AMPK, several other stress-related signaling molecules such as PERK and MAPK play a role in regulating autophagy (Bhattacharya and Eissa, 2015).

In recent years, the association between autophagy and various physiological and pathological processes has gained widespread attention. This is underscored by the ability of ATGs to interact with numerous overlapping intracellular signaling pathways that play a role in health and disease (Ktistakis and Tooze, 2016). Numerous studies have provided evidence that aberrations in the autophagy pathway have been associated with a wide variety of disorders, diseases, and viral infections (Choi et al., 2013). Furthermore, previous studies have shown that age-related declines in autophagy may contribute to the progression of certain neurodegenerative diseases and phenotypes characteristic of aging (Martinez-Lopez et al., 2015). Thus, we propose the need for an evaluation of these autophagy associations to better understand and elucidate the mechanisms that contribute to undesirable pathologies. This in turn, will help to direct future studies and investigations aimed at targeting these pathways for the development of novel therapeutics. Accordingly, this review will focus on the diverse roles of autophagy in the context of several different viral infections as well as aging. Special emphasis will also be placed on the non-canonical pathway of secretory autophagy and its divergence from other extracellular vesicle (EV) release pathways. Finally, this review will address the aspects of autophagy and exosome research that require additional attention in the future, including more careful definition of extracellular vesicle origin, physical characteristics, and functional effects.

## Autophagy and viral infection

The function of autophagy in both adaptive and innate immunity has a complex and important role. In fact, the role of autophagy, specifically selective autophagy or xenophagy, to remove intracellular pathogens including bacteria and viruses was proposed to be one of the major stimulators of the evolution of the autophagy pathway as a whole (Deretic et al., 2013). Normal innate immunity involves the sensing of antigenic forms, including double-stranded RNA or cytosolic DNA, uncapped mRNAs, and bacterial LPS by various pattern recognition receptors (PRRs) such as RIG-I-like receptors, Toll-like receptors (TLRs), NOD-like receptors, and C-type lectin receptors. The sensing of these pathogen-associated molecular patterns (PAMPs) by PRRs ultimately leads to the upregulation of genes involved in inflammatory responses, including interferons (IFNs), proinflammatory cytokines [i.e., IL-1, IL-6, and tumor necrosis factor-alpha (TNF-α)], and chemokines (Takeuchi and Akira, 2010). While many PRRs exist within host cells to sense invading pathogens and initiate anti-microbial signaling, the autophagy pathway can be stimulated by several of these activated PRRs, and indeed, the autophagy pathway actually has its own set of PRRs called Sequestosome 1/p62-like receptors (SLRs) (Saitoh and Akira, 2010; Deretic, 2012). SLRs can recognize several additional molecular patterns associated with microbes or damaged cell membranes including membrane phospholipid modifications, galectin, and ubiquitin, which then lead to the SLR recruitment of the autophagic machinery (Deretic et al., 2013). In the case of viral infections, PRRs, such as RIG-I and TLRs, and autophagy receptors tripartite motif containing 5 (TRIM5) and p62, can detect viral components and thereby induce selective autophagy against the sensed virus (Dong and Levine, 2013; Moy et al., 2014; Jackson, 2015; Galluzzi et al., 2017).

**Figure 1:**
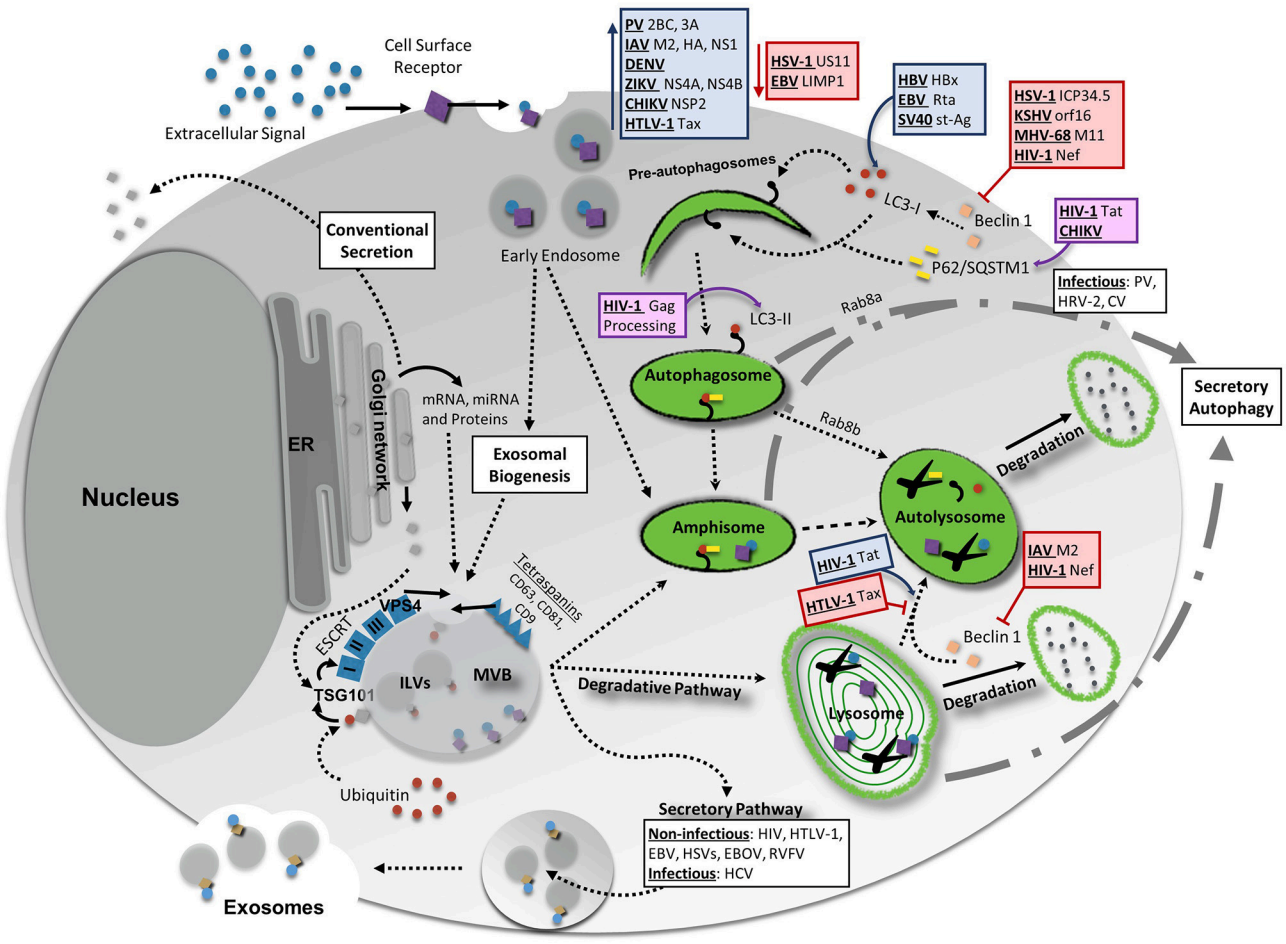
Interaction Between Vesicular Release Pathways, Autophagy, and Viral Infection. Several vesicle release pathways are utilized to maintain cellular homeostasis including the exosomal release pathway and secretory autophagy. Both pathways are capable of secreting viral products, although exosomes primarily secret non-infectious viral products with the exception of hepatitis C virus (HCV). Secretory autophagy has been shown to be responsible for the secretion of infectious particles in several cases. Degradative pathways include breakdown of select materials by fusion of autophagosomes, amphisomes, multivesicular bodies (MVBs), or endosomes with lysosomes. Up- or down-regulation of one of these pathways could potentially have feedback into other vesicular release or degradative systems to maintain cellular equilibrium. Degradative autophagy plays pro-viral and anti-viral roles during infection at various stages of autophagy. Red lines indicate a decrease/inhibition of autophagy by listed viral proteins, whereas blue arrows indicate a virally-induced increase or upregulation of autophagy. Viral proteins and viruses in purple boxes are targeted for degradation by the indicated portion of the autophagy pathway. For more detailed information, please see the main text. HIV-1, human immunodeficiency virus 1; CHIKV, chikungunya virus; HBV, hepatitis B virus; EBV, Epstein-Barr virus; SV40, simian virus 40; PV, poliovirus; IAV, influenza A virus; DENV, dengue virus; ZIKV, Zika virus; HTLV-1, human T-cell leukemia virus 1; HSV-1, herpes simplex virus type-1; KSHV, Kaposi's sarcoma-associated herpesvirus; MHV-68, murine gammaherpersirus 68.

There can be multiple end results of the induction of autophagy to oppose viral infection. The breakdown of cytoplasmic viral components or entire virions, also known as virophagy, is the most straight-forward of these (Galluzzi et al., 2017). However, other outcomes can include activation of endosomal TLRs through delivery of viral parts to endolysosomal compartments to initiate antiviral signaling cascades, modulation of reactive oxygen species production and mitochondrial stability, stimulation of cell survival, and presentation of processed viral antigens by both major histocompatibility complex (MHC) class I and II molecules to initiate adaptive immunity (Deretic et al., 2013; Dong and Levine, 2013). All of these canonical antiviral functions of autophagy aid in instilling an overall antiviral state in the host and ridding the host of the invading pathogen. Nevertheless, many viruses have evolved to thwart these inhibitory functions and hijack autophagy components for their benefit (Figure 1). In general, viruses that disrupt autophagy can fall under three main categories or a combination thereof: (i) those that replicate better by inhibiting autophagy; (ii) those that utilize the autophagy machinery in order to promote their replication; or (iii) viruses that use components from the autophagy pathway to aid in viral egress and exit (Jackson, 2015). Differences also exist between viruses that have an RNA vs. a DNA genome, which will be discussed herein.

### Autophagy and RNA viruses

Most RNA viruses fall under the category of pathogens that utilize autophagy machinery to promote replication within host cells, and some even use autophagic membranes to support their exit from the host cell (Paul and Münz, 2016). Poliovirus (PV) was the first virus of this later type to be described in 1965, when it was observed by electron microscopy to accumulate in double-membraned vesicles (Dales et al., 1965). Later, it was discovered that the expression of both the 2BC and 3A proteins of PV induced formation of the autophagic double membrane structures, which allowed for the maturation and non-lytic release of the virus (Suhy et al., 2000; Jackson et al., 2005; Taylor and Kirkegaard, 2007; Richards and Jackson, 2012). These vesicles were dependent upon ATG12 and LC3 for their formation and were positive for late endosome/lysosomal marker LAMP1 (Jackson et al., 2005). More on viral release through use of the autophagy pathway will be discussed later.

While it may seem counterintuitive, many RNA viruses in addition to PVs find it beneficial to stimulate the induction of autophagic responses in host cells. Influenza A virus (IAV) has been well documented to promote formation of autophagosomes with its M2, hemagglutinin (HA), and NS1 proteins; however, fusion of autophagosomes with lysosomes is first inhibited by the IAV M2 protein binding to Beclin-1 (Gannag et al., 2009; Zhirnov and Klenk, 2013). Inhibition of maturation of the autophagosomes while also accumulating double membraned structures is thought to prevent host cell apoptosis and allow more efficient viral replication without chopping of viral components as they are produced (Zhirnov and Klenk, 2013). This type of strategy is also exemplified by Flaviviruses such as dengue virus (DENV), Zika virus (ZIKV) and chikungunya virus (CHIKV). DENV replication, translation, and even entry into host cells has been tied to autophagy-related processes (Heaton and Randall, 2011). Many groups have shown that DENV may induce autophagy and formation of both autophagosomes as well as structures called amphisomes, which are formed from the fusion of autophagosomes with endosomes or multivesicular bodies (MVBs), and which can then act as sites of viral replication. Furthermore, generation of energy for DENV replication is obtained through the autophagy of lipids (lipophagy), emphasizing the essential role of autophagy in the DENV life cycle (Panyasrivanit et al., 2009; Heaton and Randall, 2010, 2011; Jackson, 2015). In the case of ZIKV, autophagosomes have been found to accumulate in infected cells, allowing for increased viral replication. This accumulation of autophagic vesicles has been tied to an inhibition of Akt-mTOR signaling by ZIKV NS4A and NS4B proteins, which stimulate the induction of autophagy. It was interestingly found that these actions by ZIKV may hinder normal neurogenesis in neural stem cells, thus potentially resulting in the microcephaly phenotype seen in cases of disease (Liang et al., 2016; Chiramel and Best, 2018). CHIKV, another related Flavivirus, is targeted for autophagic degradation by p62, but at the same time the viral NSP2 protein binds to the host autophagy receptor NDP52 to promote viral replication through acting as a scaffold for the viral replication machinery (Judith et al., 2013).

Measles (MeV) has an interesting relationship with autophagy and can induce the degradative pathway in one or two successive waves during infection, depending upon the virulence of the strain, to result in both pro- and anti-viral effects (Rozières et al., 2017). Early and transient induction of autophagy by MeV during the first ~1.5 h of infection is observed in non-virulent strains, whereas a second, prolonged wave of autophagy is seen in all strains of MeV starting at ~9 h post infection (Richetta et al., 2013; Rozières et al., 2017). The early wave of autophagy induction by attenuated MeV is thought to be triggered during entry via viral interactions with the host CD46/Golgi Associated PDZ and Coiled-Coil Motif Containing (GOPC)-dependent pathway, which activates Beclin-1-mediated autophagosome formation (Naniche et al., 1993; Meiffren et al., 2010). Virulent strains to do not interact with CD46, and therefore this may be an anti-viral mechanism employed by the cell to rid itself of the invading pathogen (Richetta et al., 2013; Rozières et al., 2017). Alternatively, it has been postulated that MeV utilizes this early burst of autophagy to facilitate its own replication in a manner similar to other RNA viruses. This has been shown to be the case for the wave of autophagy that takes places at ~9 h post infection, as this second degradative induction has pro-replication effects for MeV. Several autophagy-associated proteins including, NDP52, IRGM, p62, UVRAG, and T6BP, are targeted by different viral proteins (C, N, and V, mainly) and may be involved in the upregulation of autophagic pathways (Grégoire et al., 2011; Petkova et al., 2017). Ultimately, a pro-viral state is induced in the cells by these mechanisms, including a decreased innate immune response and prolonged life in infected cells (Richetta et al., 2013). However, anti-viral effects employed by p62 targeting of MeV are observed as well (Petkova et al., 2017).

Other RNA viruses, such as hepatitis C virus (HCV) and human immunodeficiency virus (HIV), induce autophagy through different mechanisms, including targeting of IRGM (immunity-associated GTPase family M) autophagy-associated protein (Grégoire et al., 2011). HCV uses three proteins (NS4B, NS5A, and NS5B) to signal autophagy, which is required for the beginning steps of viral replication (Guévin et al., 2010; Su et al., 2011; Shrivastava et al., 2012; Jackson, 2015). Induction of autophagy by HCV in this manner also leads to viral immune subversion by preventing IFN induction by RIG-I (Ke and Chen, 2011; Wang and Ou, 2015). HIV, on the other hand, has a complicated and controversial relationship with autophagy, involving both stimulatory and inhibitory actions, both of which depend on both the cell type and the stage of infection (Jackson, 2015). In early infection, HIV replication is greatly aided by autophagy, which facilitates the processing of Gag proteins to form mature virions in macrophages (Kyei et al., 2009). In neurons, the HIV-encoded Transactivator of transcription (Tat) has been found to interact with LAMP2A to elicit a dose-dependent decrease in autophagosome markers (LC3-II and SQSTM1/p62). This was linked to a promotion of autophagy progression, as well as a degradation of cellular proteins important for neuronal function (Fields et al., 2015). It should be noted that the concentrations of Tat utilized for the experiments described above were much higher than those normally found in combined antiretroviral therapy (cART)-treated patients, and therefore more accurately represents acute, uncontrolled HIV infection. The observed Tat-mediated upregulation of autophagy within neurons was also correlated with age, as HIV-infected individuals over the age of 50 were found to have markedly reduced autophagic machinery, potentially due to long-term antiretroviral therapy. Alternatively, those under 50 had elevated levels of autophagy compared to healthy controls (Fields et al., 2015). In glial cells, BAG3, a mediator of autophagy involved in autophagosome formation, was also found to be induced by the presence of Tat, further implicating Tat as an autophagy activator (Bruno et al., 2014). The autophagy stimulating properties of Tat suggest that it contributes to cell survival, and consequently, the establishment of a viral reservoir, especially in the central nervous system (CNS), during acute infection. Along these lines, it has been shown that inhibition of autophagy in T-cells can likewise restrict replication of the virus (Eekels et al., 2012; Jackson, 2015).

Conversely, degradative autophagy has been found to be detrimental to the virus if allowed to continue unchecked, as is evidenced by the targeted degradation of the HIV Tat protein by p62/SQSTM1 in CD4^+^ T-lymphocytes (Sagnier et al., 2015). Additional research has shown that long-term non-progressor patients have higher levels of autophagy markers, and that selective degradation of HIV Tat protein in CD4^+^ T-cells reduces viral replication (Nardacci et al., 2014; Sagnier et al., 2015). As a potential counter to this, the HIV protein Nef inhibits maturation of autophagosomes in macrophages by interacting with Beclin-1 and preventing lysosomal fusion, similar to the M2 protein of IAV (Kyei et al., 2009; Dinkins et al., 2015). Other studies have shown that Nef is responsible for the suppression of autophagy within astrocytes by a similar mechanism, as measured by the accumulation of autophagy marker proteins, LC3 and SQSTM1/p62 (Saribas et al., 2015). Suppression of autophagy has also been described in neurons isolated from brains of HIV-infected individuals post mortem, and that inhibition of autophagy as a result of HIV infection was correlated with neuronal cell death and accompanying clinical neurodegeneration (Alirezaei et al., 2008). Despite the overall observed decrease in autophagy, transient increases in autophagy were also noted in these studies. The presence of these conflicting results on the utility of autophagy induction to productive HIV infection has led to various hypotheses, including a model proposed by Zhou et al. in which HIV generally downregulates autophagy in cell types that are permissible to infection to promote viral replication, but viral products promote autophagy in cells that are not permissible to HIV in order to encourage cell survival (Zhou et al., 2011). Regardless, HIV undoubtedly has an intricate connection with autophagy which will likely be the focus of many future investigations.

Viruses that cause cancer also tend to impact autophagy, which is perhaps not surprising, as autophagy is often found to be dysregulated in cancer cells. Specifically, induction of autophagy has been cited as a mechanism to promote cell survival and prevent apoptosis; conversely, prevention of autophagy in cells undergoing oncogenesis has been postulated to prevent degradation of cancer signaling molecules and damaged protein aggregates and organelles, which ultimately contribute to the tumor growth (White et al., 2015). As such, a wide variety of inhibitors or inducers of autophagy have been formulated and are commonly used for the treatment of many types of cancers (Levy et al., 2017). Human T-cell leukemia virus (HTLV), an oncogenic retrovirus, is a good example of a virus that causes an autophagy-inducing type of cancer. The HTLV Tax protein has been found by several groups to promote and interact with the autophagy pathway in infected CD4^+^ T-cells by multiple mechanisms, including interaction with Beclin-1, prevention of autophagosome-lysosomal fusion, upregulation of Bcl-3 (which promotes autophagy), and interactions with NFκB and signal transducer and activator of transcription 3 (STAT3), ultimately resulting in an abundance of autophagosomes and promotion of viral replication (Cheng et al., 2012; Tang et al., 2012; Wang et al., 2013; Chen L. et al., 2015; Ren et al., 2015).

### Autophagy and DNA viruses

In contrast to most RNA viruses, many DNA viruses including herpesviruses, hepatitis B virus, and simian virus 40 (SV40) both interfere with different stages of autophagy pathways and induce autophagy to aid in their replication, depending upon the stage of infection. Herpesviruses were classically thought to halt autophagy, as degradation of viral products negatively impacted their life cycle. Contributing to this paradigm, one of the earliest viral genes discovered for its negative impact on autophagy signaling was the ICP34.5 protein, a neurovirulence factor important for viral replication encoded by herpes simplex virus type-1 (HSV-1) (Tallóczy et al., 2002). In the earlier stages of HSV-1 infection, autophagy is induced by the host for the xenophagic degradation of the virions as well as viral gene products. This process was shown to be antagonized by ICP34.5 targeting of Beclin-1 (Tallóczy et al., 2006; Alexander et al., 2007; Alexander and Leib, 2008), which was demonstrated by the failure of viral strains with a mutation in ICP34.5's Beclin-1 binding domain to inhibit autophagy, thus resulting in a reduced rate of viral propagation (Orvedahl et al., 2007). Autophagy has also been implicated in antigen presentation to MHC class I and II molecules for recognition by CD4^+^ T and CD8^+^ T-cells, respectively (reviewed in Crotzer and Blum, 2009). It has been recently shown that dendritic cells possess an increased capacity to present viral antigens on MHC class I molecules when they are infected with HSV-1 strains absent the ICP34.5 protein, indicating an important role for ICP34.5 in escape of the virus from immune recognition by dysregulating the autophagy (Budida et al., 2017). Other herpesvirus proteins with the ability to bind to Beclin-1 and inhibit autophagy include orf16 encoded by Kaposi's sarcoma-associated herpesvirus (KSHV) and M11 encoded by murine gammaherpersirus 68 (MHV-68) (Ku et al., 2008; Su et al., 2014). In addition to ICP34.5, HSV-1 also encodes another viral protein called US11, which can directly bind to protein kinase R (PKR) and inhibit autophagy (Lussignol et al., 2013). Other herpesvirus proteins capable of preventing autophagy include TRS1 of human cytomegalovirus (HCMV) and vFLIP of KSHV (Lee et al., 2009; Chaumorcel et al., 2012). However, while prior evidences indicated that herpesviruses in general prefer to inhibit autophagy, more recent data has implicated additional pro-viral roles for autophagic pathways. For example, the production of several herpesviruses including HSV-1, HCMV, and Varicella-Zoster virus (VZV) has been shown to be aided by functioning autophagy (Lussignol and Esclatine, 2017). Early activation of autophagy by HSV-1 was proposed to help entry of the virus into host cells, as indicated by decreased viral titers in cells pre-treated with autophagy inhibitors (Siracusano et al., 2016). Early infection by HCMV was also shown to stimulate autophagy while simultaneously blocking autophagosome degradation (McFarlane et al., 2011; Chaumorcel et al., 2012; Mouna et al., 2016). This strategy, similar to some RNA viruses described in the previous section, has been postulated to help the virus utilize autophagic membranes for its replication (Lussignol and Esclatine, 2017), although further study will likely be required to dissect out the true role of autophagy in this scenario. Additionally, gammaherpesviruses have been shown to also induce autophagy in a stage-dependent manner. Active lytic infection of cells with Epstein–Barr virus (EBV) increases LC3-II, the membrane-bound form of LC3, and LC3-containing structures. This accrual of LC3-positive assemblies and activation of autophagy was shown to be essential for lytic viral release and was attributed to the expression of the viral protein Rta, the viral transcriptional activator (Hung et al., 2014). A similar effect was observed from the KSHV lytic transcription factor RTA, which stimulates autophagy to favor viral replication and release (Wen et al., 2010). Furthermore, during latency, the latent membrane protein 1 (LMP1) of EBV is involved in both induction and inhibition of autophagy based on the stage of cells during viral transformation (Lee and Sugden, 2008; Pratt et al., 2012). Ultimately, the current evidence indicates that autophagy plays a complicated and seemingly contradictory role in the lifecycle of herpesviruses, perhaps to negatively regulate viral entry and replication early in infection, whereas later during latency, the autophagic pathways may be hijacked by the virus to promote cell survival and viral persistence, or to aid in viral reactivation and release.

Similar to herpesviruses, specific polyomaviruses have differing relationships with autophagy. BK polyomavirus (BKV) appears to benefit from autophagy induction. One study showed that while activation of autophagy by rapamycin induces the rate of viral propagation, inhibition of autophagy by several well-defined compounds reduces infection rates, suggesting that autophagy may have a key role in productive BKV replication (Bouley et al., 2014). Importantly, the functional interplay between viral proteins and autophagy pathway could be a double-edged sword. Studies on large tumor antigen (LT-Ag) of JC Polyomavirus (JCV) in cells transformed by the virus suggest that autophagy induced by BAG3, a Bcl-2-associated athanogene (proteins involved in apoptosis resistance), plays a key role in the degradation of LT-Ag in transformed cells and suppression of viral propagation in cells susceptible for viral infection (Sariyer et al., 2012; Merabova et al., 2015). Meanwhile, Kumar and Rangarajan showed that small tumor antigen (st-Ag) expressed in cells transformed by SV40, a well-known member of polyomavirus family, activates AMPK to promote autophagy to maintain energy homeostasis during nutrient deprivation (Kumar and Rangarajan, 2009). The studies for the impacts of polyomaviruses including JCV, BKV, and SV40 on autophagy pathways and role of autophagy in their replication cycle are limited and need further characterization to determine whether these viruses mainly benefit from activation or hinderance of these pathways. On the other hand, molecular interactions between hepatitis B virus (HBV) and autophagy is well documented. Tang et al. has previously shown that HBx protein encoded by HBV induces autophagy by up-regulating expression levels of Beclin-1 protein, thereby leading to an increase in LC3 positive vacuole formation (Tang et al., 2009). Shortly after, autophagy activation was shown in cells containing actively replicating HBV, whose mechanism was attributed to the HBx protein (Sir et al., 2010). Several additional studies on autophagy and its key roles in the HBV replication cycle have been reported with the main conclusion that HBV utilizes autophagic pathways to support its own replication (Tian et al., 2011; Lazar et al., 2012; Huang et al., 2014; Cheng et al., 2017; Döring et al., 2018).

## Autophagy and aging

The interaction between autophagy and viruses, while complicated, indicates the importance of this pathway in the progression of disease. Whereas viral infections may be avoided or combated with vaccines or anti-viral drugs, all organisms are subject to deterioration of tissues and cellular processes by aging. Aging is associated with a global decline in proteolytic activity and increasing intracellular accrual of organelle and macromolecular damage. Accumulating evidence is pointing to a weakening of the autophagy pathway as a key feature of aging (Del Roso et al., 2003; Matecic et al., 2010). Given the importance of the autophagy pathway in cellular maintenance, the progressive deterioration and reduction in this pathway with age is considered to have an instrumental role in the decline of aging biological systems. In fact, numerous evidences indicate that malfunction in the autophagy pathway contributes to many common age-associated pathologies, whereas activation of this pathway has beneficial effects in many models of human diseases. A simple example is lipofuscin, a brown-yellow, electron-dense, autofluorescent pigment that accumulates progressively over time in lysosomal compartment of post-mitotic cells in several tissues, such as neurons, heart and skeletal muscles (Terman et al., 2010). Although the exact mechanisms behind this accumulation are still unclear, a reduced ability of lysosomes to fuse with autophagic structure and degrade cargo has been observed with the accumulation of lipofuscin (Chuang et al., 2014). Other commonly observed age-associated pathologies coinciding with the integrity of the autophagosomal-lysosomal network includes a plethora of neurodegenerative diseases such as Parkinson's (Geisler et al., 2010; Narendra et al., 2010; Burman et al., 2012), Alzheimer's (Spilman et al., 2010), and Huntington's disease (Ravikumar et al., 2004). In addition, several age-associated metabolic syndromes including glucose intolerance, decreasing muscle mass, accumulation of lipids within tissues, and cardiovascular dysfunction have also been closely linked with autophagy (Mizushima et al., 2008; Choi et al., 2013).

Studies have shown a downregulation of the autophagy genes ATG5, ATG7, and Beclin-1 with decreased protein expression in the brains of aging populations as compared with those that were younger (Lipinski et al., 2010). Likewise, the IP3 (inositol 1,4,5-trisphosphate) receptor signaling that is responsible for Ca2^+^ release from the endoplasmic reticulum is upregulated in age-related diseases, including Alzheimer's disease (AD) and cardiac hypertrophy. This suggests that autophagy may be reduced in these conditions (Decuypere et al., 2011). Similarly, a NAD-dependent deacetylase involved in the regulation of autophagy called Sirtuin1 is downregulated during insulin resistance and metabolic syndrome (de Kreutzenberg et al., 2010). Another study showed that ULK1, Beclin-1, and LC3 downregulation was linked to osteoarthritis, a disease commonly found in the aging population (Caramés et al., 2010). A microarray-based genetic screening for factors involved in aging in *Saccharomyces cerevisiae* led to the identification of 117 mutations in 10 ATG genes leading to autophagy defects (Matecic et al., 2010). In addition, the introduction of loss-of-function mutations in several autophagy proteins including ATG1, ATG7, ATG18, and Beclin-1 ultimately decreased the lifetime of *Caenorhabditis elegans* nematodes (Tóth et al., 2008). Likewise, the life span of *Drosophila melanogaster* was significantly reduced by decreased expression of the autophagy-related proteins ATG1, ATG8, and Sestrin1 (Simonsen et al., 2008; Lee et al., 2010). Loss of these protein functions resulted in several pathologies associated with aging, including muscle degeneration, mitochondrial dysfunction, triglyceride accumulation, and cardiac malfunction (Lee et al., 2010).

Since many age-related disorders are instigated by abnormal proteins, the need to develop therapeutic strategies that can target these toxic proteins for degradation or elimination may be worthwhile. In fact, drug-based or genetic modulation of autophagy to encourage the clearance of damaging protein aggregates may protect cells from toxicity. Some models of aging and neurodegenerative diseases have shown a potential benefit of inducing autophagy in cells which may lead to protection against the induction of apoptosis or necrotic cell death (Cheung et al., 2011; Tan et al., 2014). Although possible therapeutic approaches targeting autophagy with the goal of ameliorating neurodegenerative diseases and delaying aging processes are gaining more traction, there is currently no conclusively established cause-and-effect relationship between autophagy and aging, and limited evidence proving that enhancing autophagy can revert aging. However, it is well-accepted that aging individuals possess a decline in immune function, and defects in autophagy can promote this phenotype (Cuervo and Macian, 2014). It stands to reason that in the context of aging individuals with dysfunctional autophagy pathways, the impact of viral infections could be significantly enhanced, thus resulting in exacerbated morbidity and disease in aging populations. Furthermore, the connection between long-term viral infections that affect autophagy and those effects on accelerated aging in chronically-infected individuals is poorly understood and may also be of significant interest. The specific interactions between autophagy and aging should be the focus of future studies in order to gain better understanding of autophagy processes and determination of how autophagic responses may contribute to the aging phenotype, particularly in the context of viral infections.

## Secretory autophagy, EVs, and viral infection

In the previous sections we discussed the roles of canonical degradative autophagy, and how a wide variety of viruses interact with these pathways in order to disrupt or hijack autophagic machinery for their benefit. Recently, an alternative function of autophagy has been gaining more attention, both for its roles in unconventional secretion of cytosolic components and viral particles and for its cross-talk with other cellular secretory and degradative pathways (Ponpuak et al., 2015). The actions and implications of this secretory autophagy on cellular function, extracellular vesicle release, and viral life cycle will be discussed.

### Extracellular vesicles and exosomes

Extracellular vesicles (EVs) are membrane-bound nanovesicles that arise from several cellular compartments, including the plasma membrane or the late endosome. It has been well-documented that most cell types secrete EVs and the presence of EVs has been confirmed in numerous biological fluids such as blood, saliva, cerebrospinal fluid (CSF), and urine (Colombo et al., 2014; Isola and Chen, 2017). The three key types of EVs include exosomes, microvesicles, and apoptotic bodies. These vesicles are primarily distinguished by their size, the method of their biogenesis, and the expression of characteristic proteins (Hessvik and Llorente, 2018). Briefly, microvesicles range from 100 to 1,000 nm and are released directly from the plasma membrane through budding; exosomes are typically 30–150 nm and are liberated after the fusion of MVBs with the plasma membrane; apoptotic bodies are released from dying cells and are classically larger than 1,000 nm in size (Zaborowski et al., 2015; Raab-Traub and Dittmer, 2017). While EVs were initially regarded as cellular waste, recent research has highlighted EVs as critical mediators of intercellular communication. This communication is achieved via the release of cargo-containing EVs from donor cells and their uptake by recipient cells. While the cargo of EVs is cell-type specific, EVs generally contain diverse mixtures of nucleic acids, proteins, and lipids (Zaborowski et al., 2015; Abels and Breakefield, 2016).

Over the last decade there has been an increased interest in the role of exosomes in both normal and pathological conditions. As previously mentioned, exosomes range in diameter from 30 to 150 nm and are enriched in certain proteins such as tetraspanins (CD81, CD63, and CD9, among others), major histocompatibility complex (MHC) class II proteins, and the accessory proteins TSG101 and Alix, which are linked to the endosomal sorting complex required for transport (ESCRT) pathway (Andreu and Yáñez, 2014; Zaborowski et al., 2015; Abels and Breakefield, 2016). Exosome biogenesis is directly coupled to the endosomal pathway; late endosomes can mature into MVBs and intraluminal vesicles (ILVs) are formed from the inward invaginations of the MVBs. Depending on the molecular content within the ILVs, the MVBs can be targeted for fusion with either the lysosome or the plasma membrane, with the former resulting in degradation and the latter resulting in the extracellular release of ILVs, which are then termed exosomes (Hessvik and Llorente, 2018). The exact mechanisms that determine which pathway is followed are not fully understood and continues to be an active area of research (Eitan et al., 2016).

Numerous studies have demonstrated the involvement of exosomes in normal physiological processes ranging from immune system modulation, metabolism, neuronal development, and proper cardiac functioning (Robbins and Morelli, 2014; Isola and Chen, 2017). Conversely, exosomes have been implicated in the progression of many pathologies including infection, cancer, and neurodegenerative disorders (Hosseini et al., 2013; Kahlert and Kalluri, 2013; Soria et al., 2017). It has therefore been postulated that exosome release acts as an alternative mechanism for the disposal of damaged proteins or toxic materials, and that this release may be induced during conditions of intracellular stress (Eitan et al., 2016). For these reasons, there is a high demand to further study the mechanisms of EV composition and release to better understand the role that they play in health and disease.

### Secretory autophagy

Degradation of undesirable cellular components, while a main function of autophagy, is not the only autophagic pathway available to rid the cell of unwanted molecules. Secretory autophagy is a complex and poorly characterized method for the cell to remove such material. Details of this pathway have previously been extensively reviewed (Jiang et al., 2013; Ponpuak et al., 2015). Nevertheless, we will highlight the current understanding of secretory autophagy here.

Conventional secretory pathways for cellular proteins generally require an N-terminal signal peptide on the targeted protein for recognition and delivery to the endoplasmic reticulum (ER), followed by passage through the Golgi and subsequent secretion from the cell. Unconventional secretion of proteins lacking this signal peptide, however, does take place through several mechanisms, one of which is secretory autophagy (Ponpuak et al., 2015). Secretory autophagy is closely similar to the classical autophagy pathway in that many of the same factors are utilized, albeit for different ends. These factors include ULK, Beclin-1, and LC3 proteins of the ATG family (Ponpuak et al., 2015). The actual mechanism of the biogenesis of “secretory autosomes,” the released products of secretory autophagy, has not been adequately described in mammalian cells, however, in yeast there have been some advances in understanding this pathway through following the unconventional release of the Acb1 protein (Duran et al., 2010; Manjithaya et al., 2010; Bruns et al., 2011). Initially, in yeast, formation of a structure called the compartment for unconventional protein secretion (CUPS) takes places in close proximity with the ER. This CUPS formation then shapes the starting membranous structure for autophagosomes utilized in both degradative and secretory autophagy (Bruns et al., 2011). CUPS are morphologically and likely functionally analogous to the pre-phagophore structure in mammalian cells called omegosomes (Jiang et al., 2013). Cargo selection criteria for secretory autophagy is wholly unknown, and whether specific markers such as ubiquitination or particular receptors such as p62, which are often required for degradative autophagy, are required has been unexplored. However, it is known that both proteins that include or do not include a classical N-terminal signal peptide have been efficiently ejected from the cell by secretory autophagic processes (Ponpuak et al., 2015; Papandreou and Tavernarakis, 2016). The Golgi re-assembly and stacking proteins (GRASPs), which are known to function in many roles including regulation of cargo transfer from the ER to the Golgi, maintaining Golgi stacks, controlling cell cycle events, and promoting unconventional secretion of proteins (Jarvela and Linstedt, 2012), are currently the only known markers required for secretory autophagy (Nickel and Rabouille, 2009). In mammals, it has been found that GRASP55 and GRASP65 are necessary for autophagic secretion. However, GRASP55 has likewise been found to be requisite for classical degradative autophagy, indicating that it is a less specific marker for secretory autophagy as compared to GRASP65 (Dupont et al., 2011; Deretic et al., 2012). These evidences further demonstrate that the initial autophagic structures formed may be identical in origin before dedication to degradative or secretory events (Jiang et al., 2013). There are, of course, factors that differ between secretory and degradative autophagy. For example, while Rab8a was demonstrated to be critical for secretory autophagy, Rab8b has been shown to be more important for the progression and maturation of degradative autophagosomes (Dupont et al., 2011; Pilli et al., 2012; Ejlerskov et al., 2013; Ponpuak et al., 2015). It has been conjectured that TBK-1, a serine/threonine kinase, may also play a role in the determination of secretory *vs*. degradative fates via interaction with Rab8b. TBK-1, after interaction with Rab8b, phosphorylates autophagy adaptors optineurin and SLR p62, which then promotes degradation of autophagosomal contents (Jiang et al., 2013). Alternatively, Rab8a has been found to be associated with autophagosomes that do not fuse with lysosomes, and instead fuse with the plasma membrane (Dupont et al., 2011).

Apart from the classical direct release of secretory autophagosomes from the plasma membrane, additional fates do exist for secretory autophagosomes. Fusion with MVBs is possible to first create a secretory amphisome, which then may subsequently release its cargo upon uniting with the plasma membrane (Gordon and Seglen, 1988). The consequences of this hybrid vesicle release may be the presence of EVs positive for both exosomal markers and LC3 proteins, which may contain poorly characterized cargo and functionalities that are distinctly different from conventional exosomes (Ponpuak et al., 2015). Other intersections between MVB/exosomal release and autophagy have been observed, such that it is thought that when one pathway is inhibited under cellular stress, that the other is activated in order to maintain homeostasis, much like an equilibrium (Baixauli et al., 2014; Ojha et al., 2017). Moreover, crosstalk between autophagy and EV biogenesis has been linked with several disease pathologies, including viral infection, Alzheimer's, Crohn's disease, and osteoporosis (Papandreou and Tavernarakis, 2016). Certainly, there is considerably more to be learned about the interactions and functional differences between degradative autophagy, secretory autophagy, and EV biogenesis in many contexts.

### Effect of viruses on secretory autophagy, EVs, and pathogenesis

Several viruses are able to directly use secretory autophagy as a means to exit host cells with relative ease, particularly in the case of enteroviruses. PV, human rhinovirus 2 (HRV-2), and coxsackievirus (CV) are examples of positive-sense RNA enteroviruses that exit cells through use of autophagosomes (Münz, 2017; Mutsafi and Altan-Bonnet, 2018). These viruses, although they require intracellular membranes for replication, are characterized by non-enveloped mature virions. As such, they were once thought to exit cells through lysis. Recently, it has become apparent that they utilize secretory autophagy pathways for viral release (Bird et al., 2014; Chen Y. H. et al., 2015; Mutsafi and Altan-Bonnet, 2018). A strong evidence for this was found in the case of PV, where autophagosomes (LC3-II-positive) containing viral particles were visualized to fuse with the cell membrane and release a vesicle enclosed by a single-membraned (inner membrane of the double membraned autophagosome) and containing approximately 19 virions on average (Chen Y. H. et al., 2015). Similar routes of exit have likewise been seen with CV and HRV-2 (Robinson et al., 2014; Chen Y. H. et al., 2015). The advantages of this viral release within large vesicles from cells likely includes protection of the virus from host immune defenses, as well as the ability to utilize phosphatidylserine-enriched autophagosomal membranes, which aid in efficient uptake in recipient cells (Chen Y. H. et al., 2015; Münz, 2017).

As previously mentioned, EVs have been found to contribute to many types of pathogenesis, and several studies have demonstrated that infected cells secrete exosomes containing pathogenic products. With respect to viral infection, viruses that cause chronic and persistent infections, as well as oncogenic viruses, are well known to have a strong influence on EV content (Eitan et al., 2016; Raab-Traub and Dittmer, 2017). Some examples of viruses that use exosomes for the secretion of non-infectious proteins or nucleic acids are HIV, HTLV, EBV, HCV, DENV, Ebola virus (EBOV), Rift Valley fever virus (RVFV), and herpesviruses. It is believed that in some of these cases, such as with HIV and herpesviruses, that these viruses allow the release of exosomes containing viral components to prime distant recipient cells, which thereby enhances their susceptibility to infection (Chahar et al., 2015; Anderson et al., 2016; Sampey et al., 2016; Raab-Traub and Dittmer, 2017). On the other hand, the packaging of viral components into exosomes also may mediate recipient cell damage and death, particularly in immune or CNS-resident cells, such as in the cases of EBOV and HIV (Lenassi et al., 2010; Pleet et al., 2016, 2018). It has also been shown that exosomes released from hepatitis A virus and hepatitis C virus-infected cells have the potential to spread virions that are capable of directly infecting recipient cells (Fleming et al., 2014). In these scenarios, the overall result is the enhancement of viral spread and transmission. However, in contrast, exosomes can also act as an antiviral mediator and induce signaling to create a negative impact on viral replication within recipient cells. These seemingly contradictory effects can, and often do, coexist side-by-side during viral infections. This phenomenon is exemplified by HIV, where it has been demonstrated that antiviral responses can be initiated via certain proteins such as cyclic guanosine monophosphate–adenosine monophosphate (cGAMP) or viral RNAs that are enclosed within exosomes. The recognition and transfer of this material elicits an immune response through the induction of proinflammatory signaling cascades and this contributes to the inhibition of viral replication (Hoen et al., 2016). Unfortunately, this upregulation of proinflammatory signaling can be sustained for prolonged periods of time in latently-infected individuals, ultimately leading to chronic inflammation and exhaustion of the immune response (Narayanan et al., 2013; Sampey et al., 2016; DeMarino et al., 2018).

The exact mechanisms through which viral material is incorporated into EVs is not entirely understood. Given the fact that many viruses enter the cell via endocytosis, it has been proposed that viruses have evolved mechanisms to further manipulate or hijack this system to facilitate their movement. To illustrate this point, several studies have reported interactions among the components involved in exosome biogenesis and those involved in viral infection (Raab-Traub and Dittmer, 2017). For example, the ESCRT pathway and the Rab GTPases, which are responsible for vesicular trafficking, represent attractive targets for viral manipulation. Indeed, several viruses including HIV, HSV-1, and IAV virus have been shown to utilize these complexes to aid in their transfer (Anderson et al., 2016). Due to the serious health risks posed by these viruses, there is a dire need to further study the means by which they modulate exosomal communication to promote their transmission.

It is clear that many viruses have found benefit in utilizing host pathways for vesicular release. However, what determines which of these pathways will be targeted by the virus? It is possible that release of viral particles through secretory autophagy is an evolutionary adaptation to allow release of whole virions while evading host immune responses. Alternatively, release of viral components within exosomes may illustrate a more secondary, accidental effect of viral constituent interaction with ESCRT proteins such as TSG101. On the other hand, some of the consequences of viral product-containing exosomes in recipient cells appear highly specific and beneficial for viral pathogenesis, perhaps so much so that it seems likely that viruses have purposefully utilized this to induce a particular response in recipient cells, whether that be induction of death in immune cells or priming of target cells for future infection (Lenassi et al., 2010; Narayanan et al., 2013; Jaworski et al., 2014; Pleet et al., 2016, 2018; Sampey et al., 2016). To this end, it may be that viruses harness the benefits of multiple vesicular release pathways for different functional advantages, which may be potentially related to duration of infection and response to therapeutic intervention. The extent to which each of these is exploited by various pathogens including viruses should be further characterized to expand the current understanding of disease pathogenesis.

What a “secretory autosome” looks like once released has not been characterized. As such, it may be possible that it contains several of the same surface markers as exosomes. The reasoning for this lies in that many autophagosome double membrane structures are derived from recycled membranes already present within the cell (Bento et al., 2016; Ktistakis and Tooze, 2016). Additionally, merging of secretory autophagosomes with MVBs does occur, which once again could allow for a mixture of contents from both biogenesis pathways. It is likely, due to the different natures and mechanisms of the cargo packaged into each of these ways, that the functional consequences on recipient cells from each exosomal or secretory autophagy pathways may be quite different. Therefore, it is increasingly important to pay attention to the differences between these vesicular release mechanisms and where they overlap, in order to better characterize pathogenic and host cell responses during infection and normal physiology. The interplay between vesicular release pathways is depicted in Figure 1.

## Outstanding questions and challenges

One of the inherent challenges associated with the study of EVs is the vast heterogeneity that arises from their origin, size, and the cell type from which they are secreted. This issue is further compounded in the case of viral infection since many viruses can interfere with EV biogenesis and alter their composition. To address the issue of EV heterogeneity, several methods have been designed for the purification and isolation of separate EV populations. These techniques include combinations of ultracentrifugation, precipitation-based protocols, density gradient separation, and affinity pulldown and enrichments (i.e., by antibodies against surface markers and nanoparticle enrichment; Momen-Heravi et al., 2013; Konoshenko et al., 2018). However, many of these strategies have questionable ability to truly separate out subpopulations of vesicles from others, as many of the characteristics targeted for a single type of desired vesicle can be shared by other EVs. For instance, separation of vesicles on density gradients (such as iodixanol) into fractions was once thought to allow for precise division of EV subpopulations; however, it is now known that sizes and densities of various vesicles will overlap. Exosomes are known to have floatation densities of 1.08–1.22 g/mL (Raposo et al., 1996), while vesicles isolated from the Golgi have densities of 1.05–1.12 g/mL and those from the endoplasmic reticulum sediment at 1.18–1.25 g/mL (Raposo et al., 1996; Théry et al., 2006; Momen-Heravi et al., 2013; Taylor and Shah, 2015; Konoshenko et al., 2018). To complicate this matter, some specific markers for exosomes such as tetraspanins, which supposedly differentiate exosomes from the other types of secreted vesicles, may not be as specific as previously thought. CD63 and other tetraspanins have been observed on vesicles indistinguishable from exosomes that originated from budding from the cell membrane (Booth et al., 2006; Fang et al., 2007; Lenassi et al., 2010; Andreu and Yáñez, 2014). A potential explanation for this phenomenon may be through the merging of secretory autophagosomes and MVBs, as CD63, while thought of classically as an exosomal marker, is actually a resident lysosomal protein (Robinson and Bonifacino, 2001; Blott and Griffiths, 2002). Therefore, any vesicles, including amphisomes or autophagosomes potentially released after fusion with lysosomes (Zhang and Schekman, 2013), could contain this marker. To attempt to counter these challenges, several modifications of the aforementioned techniques have been developed. A variation of ultracentrifugation that includes a simple step-wise centrifugation protocol to isolate subpopulations of EVs has been previously outlined (Théry et al., 2006). The resulting EV fractions are termed 2K, 10K, and 100K based on their sedimentation speed, with the denser vesicles being associated with the 2K, and less dense vesicles enriched in the 100K fractions. Not surprisingly, the level of expression of several exosome markers, such as tetraspanins, MHC class II, and heat shock protein 70 (HSP70) has been found to vary among these fractions, a finding that undoubtedly correlates to functional differences in recipient cells (Kowal et al., 2016). The effects of each of these vesicle subpopulations and the origin of each are still not well characterized, and it is likely that further purification will be required to isolate specific homogenous EVs of a particular derivation away from other overlapping vesicles. Careful attention must be given to the purification and characterization of the vesicles that are released and isolated by various means in order to begin to address some of the unanswered questions surrounding the connections between secretory autophagy and the EV pathways. At the very least, this characterization should include size, analysis of surface marker expression, and a profiling of the molecular cargo within the vesicles. While EVs expressing both exosome markers and autophagy-related proteins have previously been detected, to the best of our knowledge, the expression of autophagy markers among different EV fractions (i.e., 2K, 10K, and 100K) originating from the same sample have not been well characterized. Therefore, the differentiation of physical and functional characteristics between exosomes, secretory autosomes, or other types of EVs will likely be of great importance in coming years, especially in the case of viral infections.

An added layer of complexity of these unanswered questions involves the use of drugs in patients in clinic. As mentioned briefly, various cancer treatments utilize autophagy-modulating compounds to control tumor development (Levy et al., 2017). Furthermore, antiviral treatments may have unforeseen consequences impacting autophagy and/or EV pathways. Along these lines, new evidence suggests a commonly used component of cART, a nucleoside reverse transcriptase inhibitor, zidovudine (AZT), was found to induce depletion of mitochondrial DNA which resulted in increased autophagy inhibitor activity (AKT/mTOR) and decreased autophagy activator proteins. These dysregulations caused an accumulation of autophagosomes and proteins commonly degraded through the autophagy pathway (Santos-Llamas et al., 2018). Although these outcomes were observed in parenchymal liver cells, AZT can penetrate the blood-brain barrier and is found in CSF at concentrations ranging from 0.12 to 0.41 μmol/mL (Ene et al., 2011). This suggests that cART treatment could potentially contribute to the disruption of autophagy in the CNS. This was recently confirmed in an abstract published by Cheney et al. which showed increased levels of LC3-II (between 1.5- to 1.7-fold) following treatment with Tenofovir, Emtricitabine, and Truvada (cART drugs) and a concurrent 25% decrease in SQSTM1/p62 levels in macrophages. Similarly, they demonstrated a 3-fold decrease in LC3-II and a 50% reduction in SQSTM1/p62 levels in astrocytes post-cART treatment, indicating an initial increase in autophagy followed by quick downregulation (Cheney et al., 2017). Furthermore, other viral infections that do not have specific inhibitors are commonly treated with the use of IFN, typically IFN-α. It has been shown that IFN-α can induce autophagy by promoting LC3-I conversion to LC3-II, and thereby contributes to the formation of autophagosomes. Therefore, IFN-α plays an additional antiviral role by encouraging the degradation of invading viral components and antigen presentation (Schmeisser et al., 2014). Of interest, treatment of HIV-infected cells with either cART or IFN has also been shown to drastically change the number, size, and primarily contents of EVs released, further demonstrating the intricate cross-talk between these pathways geared toward removal of unwanted materials (DeMarino et al., 2018). Collectively, dysregulated autophagy pathways are not only impacted by the initial infection but could also be further manipulated by off-target effects of the therapeutics used in patients. This disorganization in the metabolic health of cells and homeostasis could potentially lead to premature aging and inflammation associated with long-term diseases and infections.

## Conclusions and future perspectives

In conclusion, while the interaction of many viruses with autophagy has been explored and the utilization of vesicular biogenesis pathways by several viruses has been characterized to some extent, the whole picture has not been well-developed in most cases. More specifically, secretory autophagy in viral infection has been very poorly described and represents a potentially important and unexplored mechanism used by viruses and other pathogens to replicate and spread. In the near future, it is likely that the importance of determining the differences and equilibrium between degradative or secretory autophagy and EV biogenesis will become apparent, particularly in the cases of disturbance of homeostasis, such as during aging, disease, or infection. Furthermore, distinction between the functional consequences of each pathway in various contexts on both the initial host cell and neighboring cells will be required to dissect those that contribute to harmful downstream effects, in contrast to those that have beneficial properties, as well as to identify potential therapeutic targets against various pathologies.

## Author contributions

MP, HB, MR, IS, and NE-H wrote the manuscript. MP, HB, and CD organized, edited, and revised the manuscript. CD and DP created the figures. MZ aided in conceptual formation of the project and discussions. FK contributed to the overall direction and coordination of the project.

### Conflict of interest statement

The authors declare that the research was conducted in the absence of any commercial or financial relationships that could be construed as a potential conflict of interest.
